# Supplementary material for: Modeling glioblastoma heterogeneity as a dynamic network of cell states
Source: Mol Syst Biol. 2021 Sep 16;17(9):e10105. doi: 10.15252/msb.202010105 (PMC8444284; doi:10.15252/msb.202010105)
Supplement: Supplementary file 5 — Source Data for Figure 3 [file MSB-17-e10105-s001.zip › Figure3A_sourcedata/GSEA_3065/hallmarks_state4.GseaPreranked.1623416296068/HALLMARK_INTERFERON_ALPHA_RESPONSE.html]

Details for gene set HALLMARK\_INTERFERON\_ALPHA\_RESPONSE[GSEA]

|  || Dataset | state4 |
| Phenotype | NoPhenotypeAvailable |
| Upregulated in class | na\_pos |
| GeneSet | HALLMARK\_INTERFERON\_ALPHA\_RESPONSE |
| Enrichment Score (ES) | 0.2825032 |
| Normalized Enrichment Score (NES) | 1.0196153 |
| Nominal p-value | 0.39759037 |
| FDR q-value | 0.41173577 |
| FWER p-Value | 0.999 |
Table: GSEA Results Summary

  

Fig 1: Enrichment plot: HALLMARK\_INTERFERON\_ALPHA\_RESPONSE      
 Profile of the Running ES Score & Positions of GeneSet Members on the Rank Ordered List

  

| PROBE | GENE SYMBOL | GENE\_TITLE | RANK IN GENE LIST | RANK METRIC SCORE | RUNNING ES | CORE ENRICHMENT || 1 | CD74 |  |  | 64 | 0.281 | 0.0803 | Yes |
| 2 | TXNIP |  |  | 92 | 0.254 | 0.1578 | Yes |
| 3 | CNP |  |  | 332 | 0.169 | 0.1739 | Yes |
| 4 | IFITM3 |  |  | 546 | 0.132 | 0.1820 | Yes |
| 5 | UBE2L6 |  |  | 609 | 0.125 | 0.2121 | Yes |
| 6 | STAT2 |  |  | 824 | 0.102 | 0.2106 | Yes |
| 7 | IFITM2 |  |  | 876 | 0.098 | 0.2338 | Yes |
| 8 | ISG15 |  |  | 884 | 0.097 | 0.2639 | Yes |
| 9 | BST2 |  |  | 953 | 0.092 | 0.2825 | Yes |
| 10 | MVB12A |  |  | 1203 | 0.076 | 0.2670 | No |
| 11 | PSME1 |  |  | 1273 | 0.072 | 0.2790 | No |
| 12 | LAP3 |  |  | 1570 | 0.059 | 0.2506 | No |
| 13 | NUB1 |  |  | 1688 | 0.056 | 0.2497 | No |
| 14 | SAMD9L |  |  | 1929 | 0.049 | 0.2268 | No |
| 15 | IFI35 |  |  | 2001 | 0.047 | 0.2305 | No |
| 16 | TRIM5 |  |  | 2003 | 0.047 | 0.2454 | No |
| 17 | CSF1 |  |  | 2180 | 0.042 | 0.2307 | No |
| 18 | ELF1 |  |  | 2295 | 0.039 | 0.2250 | No |
| 19 | SAMD9 |  |  | 2296 | 0.039 | 0.2377 | No |
| 20 | TDRD7 |  |  | 2382 | 0.037 | 0.2360 | No |
| 21 | PARP9 |  |  | 2683 | 0.031 | 0.1978 | No |
| 22 | IRF7 |  |  | 2713 | 0.030 | 0.2029 | No |
| 23 | RIPK2 |  |  | 2812 | 0.028 | 0.1963 | No |
| 24 | PSMB9 |  |  | 3055 | 0.024 | 0.1651 | No |
| 25 | SP110 |  |  | 3144 | 0.022 | 0.1581 | No |
| 26 | USP18 |  |  | 3580 | 0.010 | 0.0914 | No |
| 27 | LGALS3BP |  |  | 3757 | 0.003 | 0.0640 | No |
| 28 | PARP14 |  |  | 3950 | -0.007 | 0.0353 | No |
| 29 | EIF2AK2 |  |  | 4256 | -0.025 | -0.0055 | No |
| 30 | ISG20 |  |  | 4361 | -0.033 | -0.0117 | No |
| 31 | ADAR |  |  | 4453 | -0.040 | -0.0133 | No |
| 32 | PSMA3 |  |  | 4817 | -0.069 | -0.0495 | No |
| 33 | IRF1 |  |  | 5157 | -0.098 | -0.0725 | No |
| 34 | LY6E |  |  | 5230 | -0.104 | -0.0506 | No |
| 35 | PSME2 |  |  | 5418 | -0.121 | -0.0418 | No |
| 36 | PNPT1 |  |  | 5531 | -0.135 | -0.0165 | No |
| 37 | B2M |  |  | 5823 | -0.188 | -0.0027 | No |
| 38 | CD47 |  |  | 5968 | -0.227 | 0.0474 | No |
Table: GSEA details [plain text format]

  

Fig 2: HALLMARK\_INTERFERON\_ALPHA\_RESPONSE: Random ES distribution      
 Gene set null distribution of ES for **HALLMARK\_INTERFERON\_ALPHA\_RESPONSE**
